# Scaling and correlation properties of RR and QT intervals at the cellular level

**DOI:** 10.1038/s41598-019-40247-9

**Published:** 2019-03-06

**Authors:** Jiyeong Kim, Disheet Shah, Ilya Potapov, Joonas Latukka, Katriina Aalto-Setälä, Esa Räsänen

**Affiliations:** 1 Computational Physics Laboratory, Tampere University, Tampere, Finland; 2 Heart Group, Faculty of Medicine and Health Technology, Tampere University, Tampere, Finland; 3 Heart Hospital, Tampere University Hospital, Tampere, Finland

## Abstract

We study complex scaling properties of RR and QT intervals of electrocardiograms (ECGs) with their equivalences at the cellular level, that is, inter-beat intervals (IBI) and field potential durations (FPD) of spontaneously beating human-induced pluripotent stem cell-derived cardiomyocyte (hiPSC-CM) aggregates. Our detrended fluctuation analysis and Poincaré plots reveal remarkable similarities between the ECG and hiPSC-CM data. In particular, no statistically significant difference was found in the short- and long-term scaling exponents *α*_1_ and *α*_2_ of RR and QT intervals and their cellular equivalences. Previously unknown scaling properties of FPDs of hiPSC-CM aggregates reveal that the increasing scaling exponent of QT intervals as a function of the time scale, is an intrinsic feature at the cellular level.

## Introduction

In the last few decades, it has been shown that a healthy heart commonly exhibits fractal scaling (long-range correlations) in heart rate variability, i.e., variations in beat-to-beat RR intervals in electrocardiograms (ECGs)^1^. Alterations in the scaling properties have been observed in the case of cardiac diseases, such as congestive heart failure^2,3^, myocardial infarction^4,5^, atrial fibrillation^6^, and dilated cardiomyopathy^7^. QT intervals also exhibit spontaneous beat-to-beat fluctuations^8^ and QT variability has been an important measure in cardiac safety and drug development, because prolongation of QT intervals increases the risk of ventricular arrhythmias, such as Torsade de Pointes^9,10^, and repolarisation lability^11^. On the other hand, relatively few studies have characterised the long-range scaling properties of QT intervals under different physical conditions^12–14^.

Less is known about the beat-to-beat scaling properties at the cellular level. With the rise of human-induced pluripotent stem cell (hiPSC) technology^15^, it is now possible to study the complex non-linear properties at the cellular level. Spontaneous contraction of hiPSC-derived cardiomyocyte (hiPSC-CM) aggregates produces a field potential comparable to an ECG waveform. In particular, peak-to-peak intervals in the field potential denoted here as interbeat intervals (IBIs) correspond to RR intervals, and field potential durations (FPDs) are equivalent to QT intervals^16–20^.

Recently intrinsic power-law behaviour of IBIs of the isolated clusters of cardiomyocytes (CMs) has been characterised with one or two scaling exponents over predefined scale ranges^21–23^. In this study, we assess complex variabilities of IBI as well as FPD times series more in depth. To the best of our knowledge, scaling properties of FPDs of isolated CMs have never been investigated. Complex variabilities of RR and QT intervals of *in vivo* heart are also evaluated. Understanding the complex dynamics of IBIs and FPDs in comparison to the RR and QT variabilities is important in establishing hiPSC-CM aggregates as an ideal *in vitro* model of the human heart. Moreover, these studies provide new insights into the intrinsic QT-RR dynamics in the absence of the autonomic nervous system.

## Methods

### ECG recordings

Raw ECG recordings are obtained from the MIT-BIH Normal Sinus Rhythm database of PhysioNet^24^. RR and QT intervals are extracted using the PhysioNet algorithm^24^ and other software^25–27^. Low quality signals and ectopic beats have been discarded (see Preprocessing). The final set of ECG data contain 18 RR and QT interval time series of 24 hours from healthy individuals of 13 women (age from 20 to 50 years) and 5 men (age from 26 to 45 years). The average length of RR and QT intervals is around 9800 beats.

### Cell culture and differentiation

Oral and written information of the study has been provided and a signed informed consent has been obtained from the participants. The study has been approved by the ethics committee of Pirkanmaa Hospital District to establish, culture, and differentiate the hiPSC lines (R08070). All experiments were carried out in accordance with following all relevant rules and regulations set by Tampere University. Healthy control hiPSCs were derived from skin fibroblasts of a 55-year-old female and a 44-year-old male (hiPSC lines UTA.04602.WT, UTA.04511.WT). Both subjects showed no detectable cardiac diseases when the skin biopsy was taken. The hiPSCs were generated and characterised as described by Takahashi *et al*.^28^. The hiPSCs were cultured and differentiated as previously described^29^. All the hiPSCs were genotyped to ensure that no major cardiac genetic disease mutations were present. A normality test on Graphpad Prism 8 software (GraphPad Software, Inc., USA) was conducted on the beating frequency and field potential duration of each sample to ensure the normally distributed population.

### Multi-electrode array (MEA) measurements

Spontaneously beating cardiomyocyte (CM) aggregates (day 30–70) were manually dissected and plated on 1% gelatin-coated 6-well MEAs (Multichannel Systems, Reutlingen, Germany). Field potential signals were recorded from the CM aggregates under serum-free EB medium (knock-out DMEM, non-essential amino acids, GlutaMAX and penicilin/streptomycin) at 36 ± 1 °C at 10 kHz sampling frequency using MEA 1060-Inv-BC and MC_Rack software (Multichannel Systems, Reutlingen, Germany). The field potentials were continuously recorded for 30 minutes at the baseline. The data obtained from MEA were analysed using a custom-made analysis module in Origin2018 software (OriginLab Corporation, USA). The signals displaying the highest amplitudes, low signal-to-noise ratios, and clear repolarisation phases were chosen for the analysis.

Two parameters that were extracted were IBIs and FPDs. An IBI is defined as the time period between two consecutive depolarisation peaks. A FPD is defined as the time period measured from the first upstroke of the depolarisation wave to the baseline of the repolarisation wave. FPDs have been shown to correlate with APD90 from action potential measurements^16,20^. Both IBIs and FPDs were extracted using a custom-developed analysis module in Origin 2017 (MicroCal Origin™, USA), in which each upstroke of the depolarisation wave is detected as the start of a field potential, and the end of the field potential is calculated semi-automatically by detecting where the repolarisation decay phase intersects with the 0 μV line abscissa. The lengths of IBI and FPD time series range from 900 to 3000 beats with the average length around 1600 beats. The IBI and FPD data-sets are available from the corresponding author on reasonable request.

### Preprocessing

Each time series was filtered before analysis in order to discard any artificial noises and ectopic beats. All the RR and QT intervals or their cellular equivalences below 200 and above 3000 ms were systematically discarded as nonphysical values. Then an appropriate envelope, or lower and upper limit, was selected around a global trend, so that the intervals outside the envelope were filtered out. The trend was calculated with a 5th order polynomial fit and twice the standard deviation of the time series was used for the envelope size, or limits.

### Poincaré plot

A Poincaré plot is a standard method to measure and visualise the temporal correlation of a time series at the shortest time scale. For a given time series {*x*_*t*_}_*t* = 1, …, *N*_, each *x*_*t*_ is plotted against *x*_*t* + 1_. To characterise the distribution of the data points on the plane, an ellipse fitting technique^30^ is employed. The standard deviation of the data points perpendicular to the line *x*_*t*_ = *x*_*t* + 1_, denoted as SD1, represents short-term variability of the data^31^. The standard deviation along the line *x*_*t*_ = *x*_*t* + 1_, denoted as SD2, reflects long-term variability, implied by the relation:

1 $${\rm{SD}}{1}^{2}+{\rm{SD}}{2}^{2}=2{\sigma }^{2},$$

where *σ* is the standard deviation of the time series^31^. Equation 1 is equivalent to the statement that the sum of short-term and long-term variability is the total variability. SD1 and SD2 are computed from the eigenvalues of the covariance matrix between *x*_*t*_ and *x*_*t* + 1_.

A complementary measure to SD1 and SD2 is the Pearson’s correlation coefficient *r*, which represents the linear correlation between time series *x*_*t*_ and *x*_*t* + 1_, defined by

$$r=\frac{{\rm{cov}}({x}_{t},{x}_{t+1})}{{\sigma }_{{x}_{t}}\,{\sigma }_{{x}_{t+1}}},$$

where cov stands for the covariance and *σ* is the standard deviation. The Pearson *r* ranges from −1 to 1, where values closer to 1 indicate positive linear correlation, and values closer to −1 indicate negative correlation.

### Detrended fluctuation analysis

Detrended fluctuation analysis (DFA), originally introduced by Peng *et al*.^32^, has been established as a reliable method to detect long-range correlation in a non-stationary time series. We follow the algorithms as described by Kantelhardt *et al*.^33^. The implementation of the algorithm has been validated against the PhysioNet DFA software package^24^; see section 1 of the supplementary information for more details. For a time series of length *N* with observations ${\{{x}_{t}\}}_{t=1,\ldots ,N}$, the DFA procedure can be summarised in four steps:

1. The profile of the time series is defined by taking an integrated sum of the series:
  $$y(k)=\sum _{t=1}^{k}({x}_{t}-\langle x\rangle ).$$
2. The profile is divided into *N*/*s* non-overlapping windows of equal length *s*. In each window, an *n*-degree polynomial approximation *y*_*tr*_ representing a local trend is computed by a least-squares fit. For our analysis we use the first-order DFA, in which a linear trend is eliminated from each window. When the windows does not divide the profile evenly, a reverse ordering of the window is averaged with the original ordering, so that the windows cover the whole profile.
3. The root-mean-square of the average variance of the residuals (*y* − *y*_*tr*_) over all 2*N*/*s* windows defines the fluctuation *F* for window size *s*.
  2 $$F(s)=\sqrt{\frac{1}{2N/s}\,\sum _{m=1}^{2N/s}\,[\frac{1}{s}\sum _{i=1}^{s}{[{y}_{m}(i)-{y}_{tr,m}(i)]}^{2}]}$$
  The window sizes range from *s* = 4 to *s* = *N*/4.
4. In presence of power-law scaling, *F*(*s*) ~ *s*^*α*^. The scaling exponent *α* is the slope of *F*(*s*) in log-log scale.

The scaling exponent *α* describes the nature of the correlation present in the data. The white noise with no correlation and Brown noise are characterised by *α* = 0.5 and *α* = 1.5, respectively. Values 0.5 < *α* < 1.5 indicate long-range correlation, and values *α* < 0.5 correspond to anti-correlations. The value *α* = 1 corresponds to 1/*f* or “pink” noise, often referred to as fractal.

More than one scaling exponents may be required to describe different correlations at different scales. It is a common practice to define two scaling exponents, *α*_1_ and *α*_2_, to describe short-range and long-range correlations, respectively. It is also possible to calculate the gradient of *F*(*s*) as a function of *s* and define a spectrum *α*(*s*), also known as continuous *α* or local *α*. Here we use the *αβ* filter^34^, which is a simplified version of a Kalman filter, to recursively estimate a local least-squares fit for tracking the evolution of the gradient of *F*(*s*) in log-log scale. The *α* spectrum provides a more complete description of complex correlation properties of a given time series than two scaling exponents with predefined scale ranges. Despite possible limitations of the *αβ* filter, such as over-smoothing or under-smoothing of the gradient depending on the choice of its parameters, the method is sufficient for our purpose to assess the general scaling patterns of our time series data. The limitations may be overcome by advanced filtering techniques using other types of smoother based on Kalman filters^35^.

### Statistical analyses

Normality of the measures obtained from Poincaré analysis and DFA was checked with Shapiro-Wilk test. When comparing the measures between ECG and hiPSC-CM data Welch’s t-test was employed to determine the statistical differences. If a variable did not meet the normality requirement for t-test, non-parametric Wilcoxon rank-sum test was employed. All the measures are presented in min-max, median, and interquartile range (Q1–Q3). Distributions of the measures are plotted in section 2 of the supplementary information.

## Results

### Quantitative analysis of Poincaré plots

**Figure 1:**
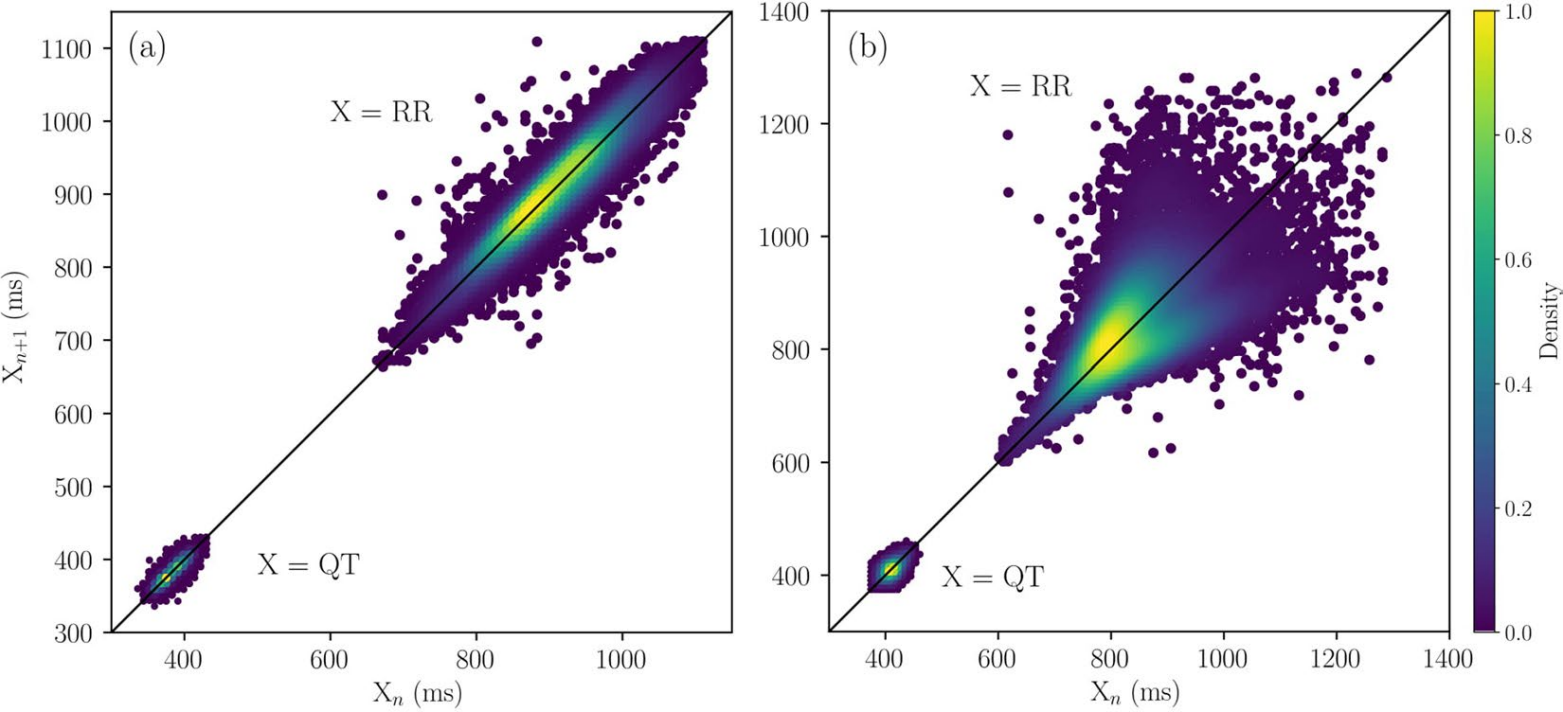
Examples of Poincaré plots of RR and QT intervals. A typical Poincaré plot for RR intervals has an elongated elliptical shape shown in (**a**). A few samples of RR interval time series has a fan-shaped plot shown in (**b**). Poincaré plots of QT intervals have smaller and rounder elliptical shapes, compared to those of RR. The density of the points is shown with a colour bar.

Figure 1 shows the representative Poincaré plots of RR and QT intervals extracted from ECGs. Most of the RR intervals show a shape of an ellipse, as in Fig. 1(a), but 20% of the samples have a fan-like shape, which is spread out towards larger RR intervals, as shown in Fig. 1(b). Results are in line with the previous studies showing the Poincaré plots of RR intervals are spread along the line of identity^36^.

The Poincaré plots of the corresponding QT intervals, also shown in Fig. 1 in the same time scale, have elliptical shapes along the line of identity, but with less eccentricity and variation, compared to those of the RR intervals. The standard quantitative measures to characterise the poincaré plots, averaged over 18 control ECG recordings, are listed in Table 1.

**Table 1:** Standard measures of the Poincaré plots of RR and QT intervals represented by min-max, median, and interquartile range (Q1–Q3). The values are obtained over *n* = 18 ECG samples, and those of IBIs and FPDs of the hiPSC-CM aggregates, obtained over *n* = 21 IBI and *n* = 20 FPD time series. p-values are computed using independent two-sample t-test.

Measures	ECG, RR (*n* = 18)	hiPSC-CMs, IBI (*n* = 21)	p-value
	min-max, median, (Q1–Q3)	min-max, median, (Q1–Q3)	
Mean (ms)	871–1830	703–2383	0.0207
	**1092**	**1400**	
	(1044–1232)	(1178–1745)	
SD1/SD2 (n.u)	0.15–0.54	0.03–0.98	0.749
	**0.28**	**0.28**	
	(0.22–0.33)	(0.16–0.45)	
Pearson’s r (n.u)	0.55–0.96	−0.02–1.00	0.483
	**0.85**	**0.85**	
	(0.80–0.91)	(0.67–0.95)	
**Measures**	**ECG, QT (** ***n*** ** = 18)**	**hiPSC-CMs, FPD (** ***n*** ** = 20)**	**p-value**
	**min-max, median, (Q1–Q3)**	**min-max, median, (Q1–Q3)**	
Mean (ms)	377–501	394–1529	<0.001
	**419**	**902**	
	(406–434)	(764–982)	
SD1/SD2 (n.u)	0.21–0.83	0.13–0.98	0.023
	**0.45**	**0.72**	
	(0.35–0.67)	0.53–0.88	
Pearson’s r (n.u)	0.19–0.92	−0.15–0.97	0.015
	**0.66**	**0.32**	
	(0.38–0.78)	(0.08–0.56)	

**Figure 2:**
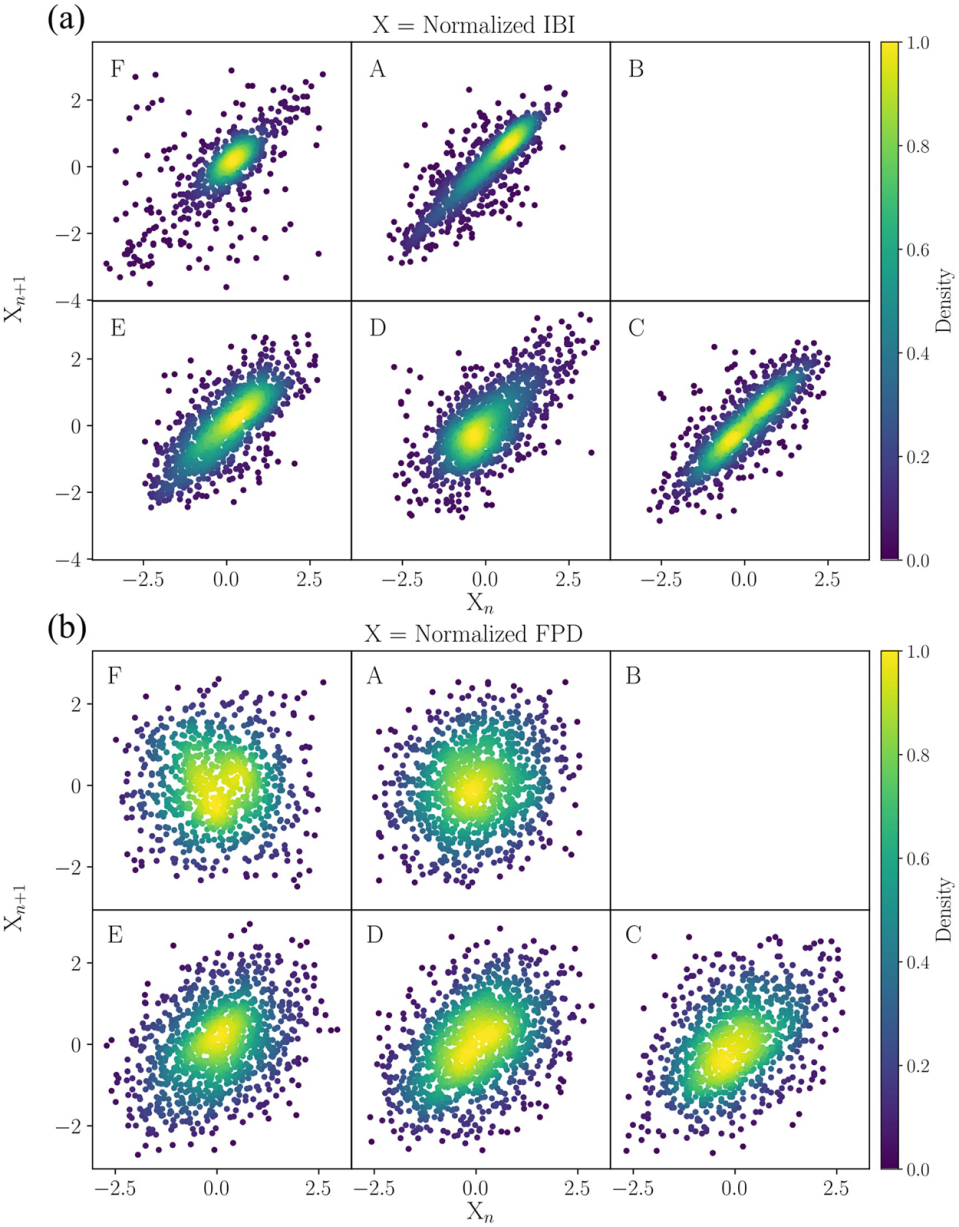
Poincaré plots of (**a**) IBI and (**b**) FPD time series extracted from the field potentials of the hiPSC-CM aggregates. The cell aggregates are plated on a six-well MEA with each well labelled as A, B, C, D, E, and F. Each Poincaré plot is centred at zero and scaled by the standard deviation. The density of the points is shown in colour.

Figure 2 and Table 1 show the Poincaré plots and the related measures of IBI and FPD time series of the hiPSC-CM aggregates, which correspond to RR and QT interval times series of ECGs. Each Poincaré plot Fig. 2(a) is plotted for the IBI time series, measured from a cell aggregate in each well, labelled as A, B, C, D, E, and F on a six-well MEA. The Poincaré plots in Fig. 2(b) are plotted for the corresponding FPD time series.

The IBI and FPD values vary considerably among the aggregates. The hiPSC-CM aggregates tend to behave more erratically as they contract spontaneously without any inputs from the autonomous nervous system. Their beat-to-beat variations are also extremely sensitive to small fluctuations in the environment, e.g., in the temperature, pressure, oxygen levels, and ion concentration. Consequently, the absolute measures of SD1 and SD2 have large variances. In order to make the visual comparison of the Poincaré plots among the aggregates easier, we have normalised the data by subtracting the mean offset and scaling by the standard deviation, so that each Poincaré plot in Fig. 2 is centred at zero and comparable with each other in magnitude. Therefore, instead of computing SD1 and SD2 separately, we examine the ratio SD1/SD2 and Pearson’s correlation coefficient *r* between the different groups, which are unaffected by the normalisation. There is a remarkable agreement between RR and IBI in SD1/SD2 and *r*, while a discrepancy exists between QT and FPD. The significantly larger SD1/SD2 (with statistical significance *p* = 0.023) and smaller *r* (*p* = 0.015) of the FPD Poincaré plots suggest that the FPD time series have significantly larger short-term variability with respect to the long-term variability.

### Detrended fluctuation analysis

**Figure 3:**
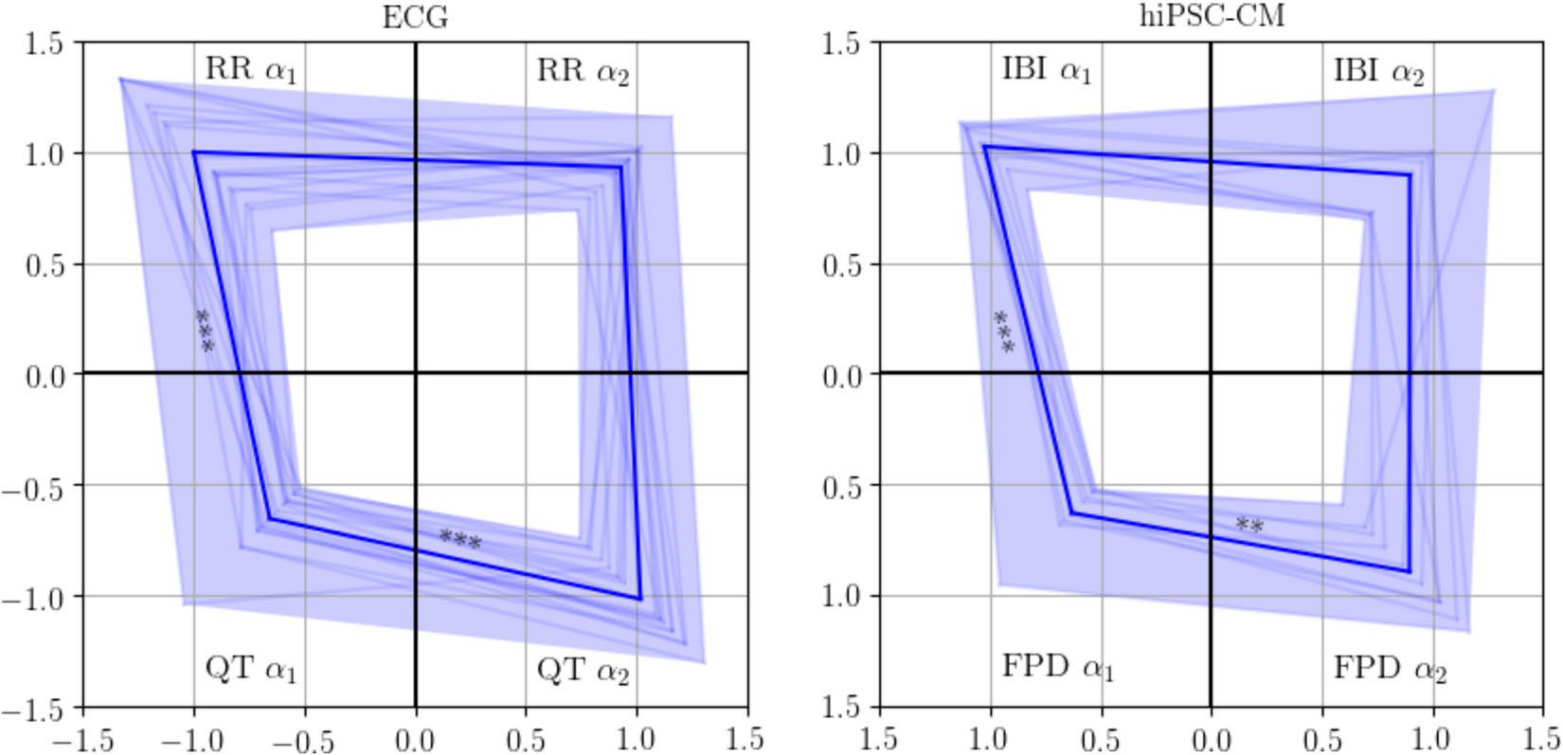
Visualisation of the DFA *α*_1_ in the time scale of beats <20 and *α*_2_ in the scale of beats >30 for ECGs (left panel, *N* = 18) and hiPSC-CM aggregates (right panel, *N* = 10), respectively. Each corner of the quadrilateral corresponds to an *α* value. The mean value is marked with bold line, and the statistical significance of the difference in the *α* values are denoted with asterisks with following significance level: *p < 0.05, **p < 0.01, and ***p < 0.001.

We first quantify two scaling exponents *α*_1_ and *α*_2_, which represent short-range and long-range correlations, respectively. In particular, *α*_1_ is calculated in the time scale of less than 20 beats, *α*_2_ in the scale of more than 30 beats. The *α*_1_ and *α*_2_ values of RR and QT intervals of ECGs and IBIs and FPDs of the hiPSC-CM measurements are visualised in Fig. 3. Each corner of the quadrilaterals corresponds to a scaling exponent. The mean *α* values are marked with bold lines and the spread of the values is shown in coloured bands. The means and standard deviations of the *α*_1_ and *α*_2_ values are also listed in Table 2.

**Table 2:** Min-max, median, and interquartile range (Q1–Q3) of the DFA scaling exponents *α*_1_ and *α*_2_ for ECGs (*n* = 18) and the hiPSC-CM aggregates (*n* = 10). Independent two sample t-tests and Wilcoxon rank-sum test are performed between *α* values of ECGs and hiPSC-CM aggregates to obtain p-values. High p-values indicate that the values are drawn from a same distribution, hence, similar.

ECG (*n* = 18)		hiPSC-CMs (*n* = 10)		p-value
	min-max, median, (Q1–Q3)		min-max, median, (Q1–Q3)	
RR *α*_1_	0.64–1.33	IBI *α*_1_	0.83–1.14	0.730
	**0.94**		**1.03**	
	(0.85–1.17)		(0.98–1.11)	
RR *α*_2_	0.73–1.16	IBI *α*_2_	0.69–1.28	0.516
	**0.94**		**0.93**	
	(0.90–0.99)		(0.72–0.99)	
QT *α*_1_	0.52–1.04	FPD *α*_1_	0.52–0.96	0.362
	**0.63**		**0.60**	
	(0.57–0.70)		(0.54–0.66)	
QT *α*_2_	0.74–1.31	FPD *α*_2_	0.59–1.17	0.090
	**1.05**		**0.91**	
	(0.89–1.15)		(0.74–1.03)	

Figure 3 allows us to compare the relative magnitudes of the *α* values from the shapes of the quadrilaterals. On the average, the quadrilateral for the hiPSC-CM aggregates resembles that of ECGs. The top sites of the quadrilaterals connecting the *α*_1_ and *α*_2_ of RR intervals and IBIs are flat, i.e., *α*_1_ ≈ *α*_2_ ≈ 1, suggesting that the fractal-like scaling property is invariant over the time scale. On the other hand, QT intervals have significantly different *α*_1_ and *α*_2_. The mean *α*_2_ is close to one, indicating that QT intervals are long-range correlated as the RR intervals, while the mean *α*_1_ is much closer to 0.5, indicating that the correlation properties resemble those of white noise in the short time scale. The scaling behaviour of FPDs is in notable agreement with that of QT intervals, with a small discrepancy in *α*_2_ (*p* = 0.09).

**Figure 4:**
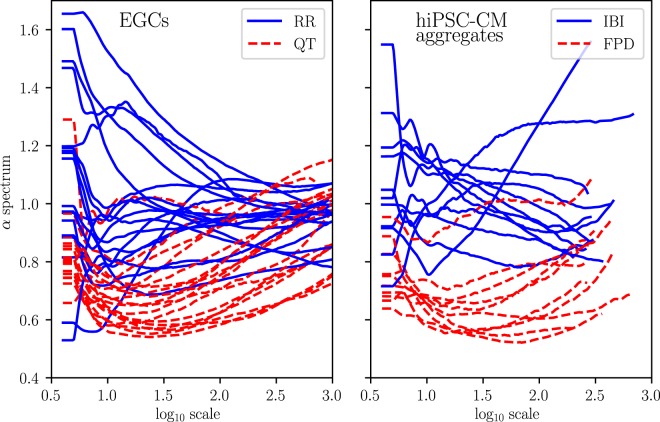
Continuous spectra of *α* of RR and QT intervals (left) and IBIs and FPDs (right) over the log scales. Local *α* s are estimated using the *αβ* filter^34^.

In further analysis, we examine the spectra of the scaling exponent *α* defined over a continuous time scale. The estimation of local *α* using the *αβ* filter^34^ produces a smooth spectrum, which depicts the evolution of the scaling exponents over the scales from short-range to long-range. The left panel of Fig. 4 shows the characteristic scaling patterns of the *α* spectra of RR and QT intervals. The *α* spectra of RR intervals approach a constant value with increasing scale resulting *α*_1_ ≈ *α*_2_ ≈ 1. In contrast, the *α* spectra of QT intervals start at lower values and increase with scale, hence *α*_2_ > *α*_1_. Similar results have been shown in the case study of the heart rate variability during pregnancy^12^.

Overall, Fig. 4 reveals notable similarities in the scaling patterns of ECGs and the hiPSC-CM aggregates. The hiPSC-CM aggregates tend to behave more erratically. Consequently, their *α* spectra are not as precise as those of ECGs. However, the scaling patterns observed in ECG, namely, the descending *α* spectra of RR intervals and the increasing *α* spectra of QT intervals, are clearly present in the spectra of hiPSC-CM aggregates. Unfortunately, the field potential measurements of the hiPSC-CM aggregates are limited in length to about 1600 intervals, so that the maximum window size in DFA is about 400 (see Methods). Therefore, we are not able to compare the spectra for larger scales than what is shown in Fig. 4. This result, however, already shows that the scaling properties in beat rates of the hiPSC-CM aggregates are well comparable to those of ECGs.

## Discussion

Our quantitative analysis reveals that RR intervals and their *in vitro* equivalent from hiPSC-CM aggregates (IBIs) share a common geometry in Poincaré plots with a positive linear correlation between two consecutive beats. Quantitative measures according to the ellipse fitting technique^30^ show that the ratio of short-term to long-term variabilities is consistent between the ECGs and the hiPSC-CM data. Therefore, clusters of hiPSC-CMs exhibit beat rate dynamics comparable to a human heart. This has also been confirmed in previous studies^21,22^. On the other hand, there is a significant difference between the hiPSC-CM aggregates and human hearts in variations of the field potential durations, i.e., QT intervals and FPDs. The ratio of short-term to long-term variability is larger in FPDs. This may be due to the erratic and immature nature of the hiPSC-CMs, causing random fluctuations between consecutive beats. However, as it did not affect the IBIs in the same way, the reason may be found in the non-trivial relationship between QT and RR intervals (see below).

The complexity of the RR and QT variabilities and their equivalences at the cellular level is further examined in their longer-range scaling properties. The conventional way to compute short- and long-term scaling exponents *α*_1_ and *α*_2_ in two predefined scales shows a remarkable agreement between RR intervals and IBIs (Fig. 3 and Table 2). The results are in line with previous reports^3,21,22^. Here, more complete descriptions of the scaling properties are given by the full spectra of the scaling exponents. The *α* spectra of RR and QT in Fig. 4 are in good agreement with results from previous studies^12,34^.

The *α* spectra of IBI and FPD, reported here for the first time, show a notable similarity with the RR and QT intervals of the ECG data. The overall resemblance implies that the distinct scaling patterns of RR and QT intervals are independent of the autonomous regulation of the nervous system, as it is also present in the isolated hiPSC-CM aggregates without any external stimulation. The *α* spectra of the FPDs stay at low scaling exponents, but the increasing trend towards larger scales–similar to that of the QT intervals–is present. The less prominent long-range correlation may be due to the different QT-RR relationship at the cellular level.

**Figure 5:**
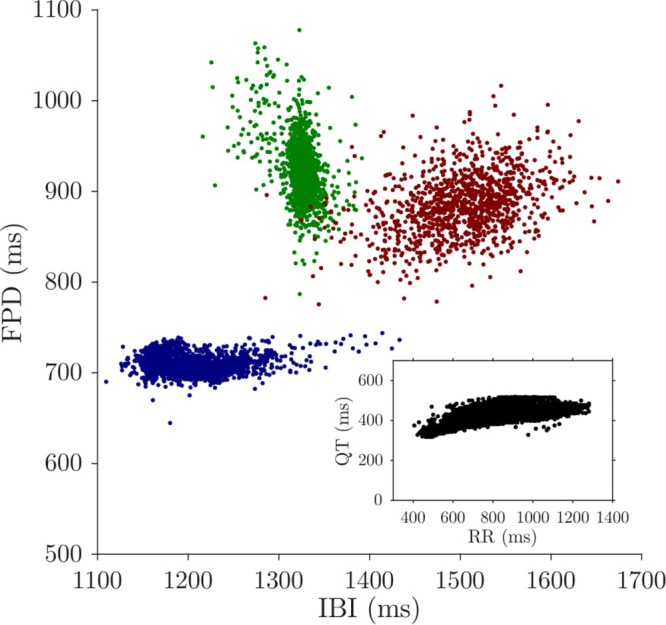
Non-trivial relationship between IBI and FPD. Each colour represents an hiPSC-CM aggregate. FPDs do not necessarily have a positive correlation with IBIs, which is found in a typical QT-RR relationship, shown in the inset.

In a normal heart, QT variability is often described in the context of heart rate variability, because the variability in RR intervals is the major physiological source of variability in QT intervals^8^. There is a clear response of QT to the acceleration and deceleration of the heart rate^37^. Moreover, recent studies have shown that there is a non-trivial dynamic relationship in terms of transfer entropy between RR and QT intervals^38^. The relationship is not as clear at the cellular level; FPDs do not necessarily have a positive correlation with IBIs, contrary to the well-known QT-RR relationship (See Fig. 5). Therefore, the discrepancies that appear only for FPDs but not IBIs, such as lower scaling exponents and relatively large short- to long-term variability ratio of FPDs, depicted by the Poincaré plots above, may be due to the less significant influence of IBIs on FPD variability compared to that of RR interval changes on QT variability.

The spontaneously beating hiPSC-CMs are relatively immature cells, which better represent a fetal heart with an underdeveloped contraction machinery and organised structural filaments, when compared to adult cardiomyocytes^39^. Even though the hiPSC-CMs have an expression of similar panel of ion channels as adult cardiomyocytes, they may have divergent expression levels of atrial, nodal, and ventricular cardiomyocytes^40^. The varying expression levels and other factors, such as differentiation techniques, age of the cells, variations in temperature and ion concentrations in the recording solutions, and recording protocols may introduce erratic variability in the beat rates of hiPSC-CM aggregates. Therefore, the interpretation of the results must be practised with caution. However, our results show that the beating dynamics of the hiPSC-CM aggregates resemble those of *in vivo* heart, despite their relative immaturity and the absence of the autonomic neural input.

Even though the hiPSC-CM aggregates were derived from only two subjects, we assume they are independent, as there are many factors in culture and differentiation process of the hiPSCs that introduce variations among cells, such as passage number, age of the cells, and batch of cultures and differentiation. After the cardiac differentiation, different cell types (nodal, atrial, and ventricular), various sizes and number of beating cells in the culture further give rise to variations among the cell aggregates.

A recent study has suggested that in the presence of inter-cellular connections along with electro-mechanical interactions, intrinsic clock-like signalling of the pacemaker cells in sinoatrial node tissues, which are similar to hiPSC-CM aggregates *in vitro*, adapts and modifies their beat dynamics, contributing to the overall fractal-like behaviour of the heart^41^. Therefore, our findings support the idea that hiPSC-CM aggregates could be an ideal *in vitro* model of a heart, i.e., a suitable platform (i) to model cardiac diseases, (ii) to screen new treatment options, and (iii) to assess cardiac safety of new chemical entities of potential new drug candidates.

Fractality and power-law behaviour of IBIs have been attributed to ion-channel gating and intra-cellular mechanisms that are themselves non-linear processes. In an adult CM, variation in intra-cellular Ca^2+^ is the main trigger for excitation-contraction coupling, which generates mechanical contraction. Due to immaturity, the Ca^2+^ transient is slower and smaller in amplitude for hiPSC-CMs^42^, but is clearly present and closely related to the beat rate variability (BRV) of hiPSC-CM aggregates. In particular, the intra-cellular sarcoplasmic reticulum (SR) Ca^2+^ cycling and mitochondrial Ca^2+^ extrusion, and the crosstalk between SR and mitochondria^43^ exhibit fractal behaviour, hence contribute to the fractal BRV at the cellular level^22,44^.

Intra-cellular Ca^2+^ cycling, among other cellular processes, also contributes to the beat-to-beat variation of the overall repolarisation, which causes variations in QT intervals at a stationary heart rate^8^. Therefore, we may postulate that the fractal behaviour of the intra-cellular Ca^2+^ cycling is also accountable for the intrinsic fractal scaling of FPDs in hiPSC-CM aggregates. There are also other factors, such as stochastic fluctuations in ion currents and inter-cellular interactions that cause variations in QT intervals. Similar effects may be present also in FPDs, which calls for further systematic investigations.

Other standard HRV time- and frequency-domain measures are available in the section 3 of the supplementary information.

## Supplementary information

Supplementary information
