# Supplementary material for: Scaling and correlation properties of RR and QT intervals at the cellular level
Source: Sci Rep. 2019 Mar 6;9:3651. doi: 10.1038/s41598-019-40247-9 (PMC6403385; doi:10.1038/s41598-019-40247-9)
Supplement: Supplementary file 1 — Supplementary information [file 41598_2019_40247_MOESM1_ESM.pdf]

Supplementary Information

Scaling and correlation properties of RR and QT intervals at the cellular level

Jiyeong Kim[1,*], Disheet Shah[2], Ilya Potapov[1], Joonas Latukka[1], Katriina Aalto-Setälä[2,3], Esa Räsänen[1]

[1]Computational Physics Laboratory, Tampere University, Tampere, Finland
[2]Heart Group, Faculty of Medicine and Health Technology, Tampere University, Tampere, Finland
[3]Heart Hospital, Tampere University Hospital, Tampere, Finland
[*]jiyeong.kim@tuni.fi

1 Validation of DFA implementation

We use publicly available MIT NSR database [1] to validate our implementation of detrended fluctuation analysis (DFA) [2] algorithm against the PhysioNet's DFA software package [1]. Using PhysioNet's algorithm and official annotations, RR and QT_{end} intervals were calculated for each recordings. Each time series was filtered with the same preprocessing procedure described in the Method section. The preprocessed RR and QT intervals were then used as inputs to our own DFA implementation and PhysioNet DFA program. In both implementations, following input parameters were used:

- linear detrending.
- minimum window size = 4.
- maximum window size = $N/4$, where N is the length of the time series.

Both implementations produce fluctuations $F(s)$ as a function of the scale s. Scaling exponents, the slope in the log-log plot, are calculated by a linear regression function, `scipy.stats.linregress`, in the scale range of 4-30 beats.

For 18 RR interval time series, mean $\pm$ standard deviation of the scaling exponents α computed with our implementation was 0.998 ± 0.142, and that of scaling exponents computed using PhysioNet DFA was 0.999 ± 0.148. Root mean square error (RMSE) and normalised RMSE (NRMSE), i.e., RMSE normalised by the mean of the PhysioNet results, were computed to make comparison between results of two implementations: RMSE = 0.01880 and NRMSE = 0.01881. Similarly, for 18 QT interval time series, our implementation yielded $\alpha = 0.693 \pm 0.126$ and PhysioNet DFA $\alpha = 0.692 \pm 0.124$, with RMSE = 0.00433 and NRMSE = 0.00626.

The minor numerical differences arise in selecting a set of window sizes or scales s, at which fluctuations $F(s)$ are computed. PhysioNet DFA selects a set of window sizes such that the ratio between successive window sizes is $2^{1/8}$ ranging from predefined minimum window size to maximum window size. On the other hand, our implementation chooses number of window sizes as an input and generates a set of window sizes that are equally spaced in log scale and round them. Therefore, as fluctuations are computed at different set of scales, numerical deviations arise when computing the slope.

An important difference between the two implementations is in handling the window sizes that do not divide the time series evenly. While PhysioNet DFA discards the end of the data that is not covered by non-overlapping windows (there is also an option of using sliding windows), our implementation follows the algorithm illustrated by Kantelhardt et al. [3], in which the time series is divided into non-overlapping windows also starting from the end so that no data is neglected. Therefore, the different procedures introduce the deviations in the fluctuation.

As supported by low RMSE and NRMSE between the results of two implementations, the numerical deviations in the results do not affect our analysis, thus showing that our implementation produces valid DFA results.

2 Distributions of the measures

We report the distributions of the measures presented in Table 1 and 2 of the manuscript. Table 1 contains Poincaré plot related measures, such as mean, ratio of SD1 to SD2, and Pearson's correlation coefficient r for RR, QT intervals and IBIs and FPDs, of which the histograms are presented in Fig. S1 - S3. Table 2 contains scaling exponents from DFA. Histograms of scaling exponents α_1 and α_2 of RR, QT, IBI, and FPD are presented in Fig. S4.

Distributions are checked in order to make a valid comparison of the presented measures between ECG and hiPSC-CM data. The variables from IBI and FPD have higher variances in general compared to RR and QT. That is, equal variance is not satisfied between compared variables; therefore, Welch's t-test has been employed. Normality of the variables were also checked with Shapiro-Wilk test. When the normality requirement is not met, instead of t-test, non-parametric Wilcoxon rank-sum test was used.

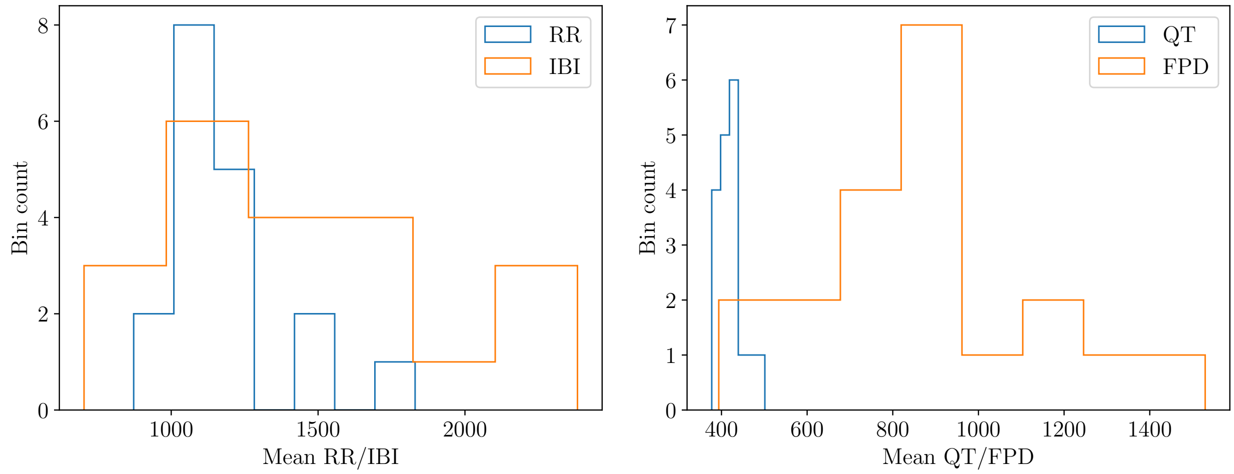

Figure S1: Distribution of mean RR / IBI (left) and mean QT / FPD (right).

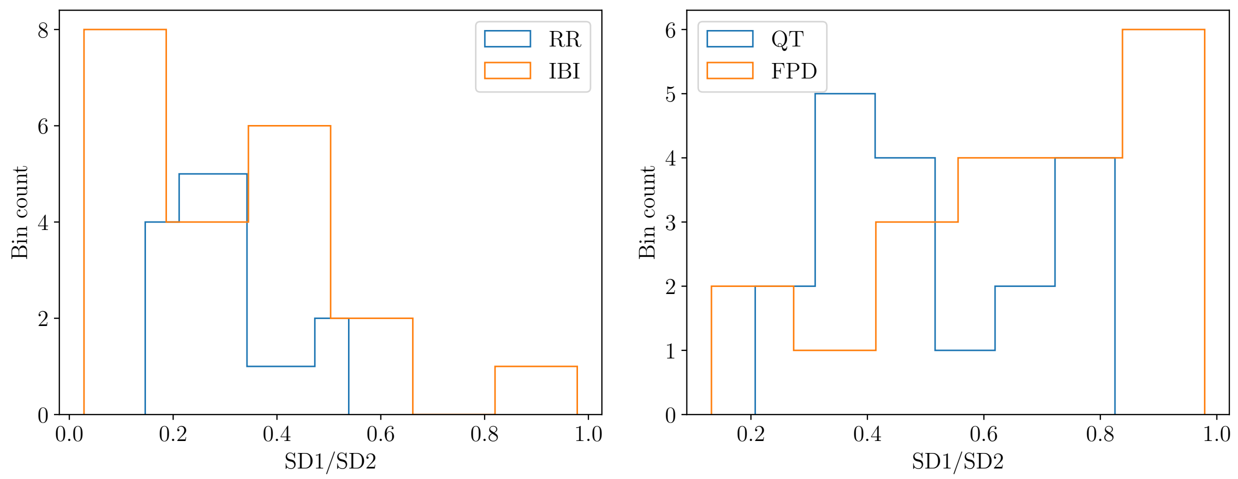

Figure S2: Distribution of the SD ratio (SD1/SD2) of RR / IBI (left) and that of QT / FPD (right).

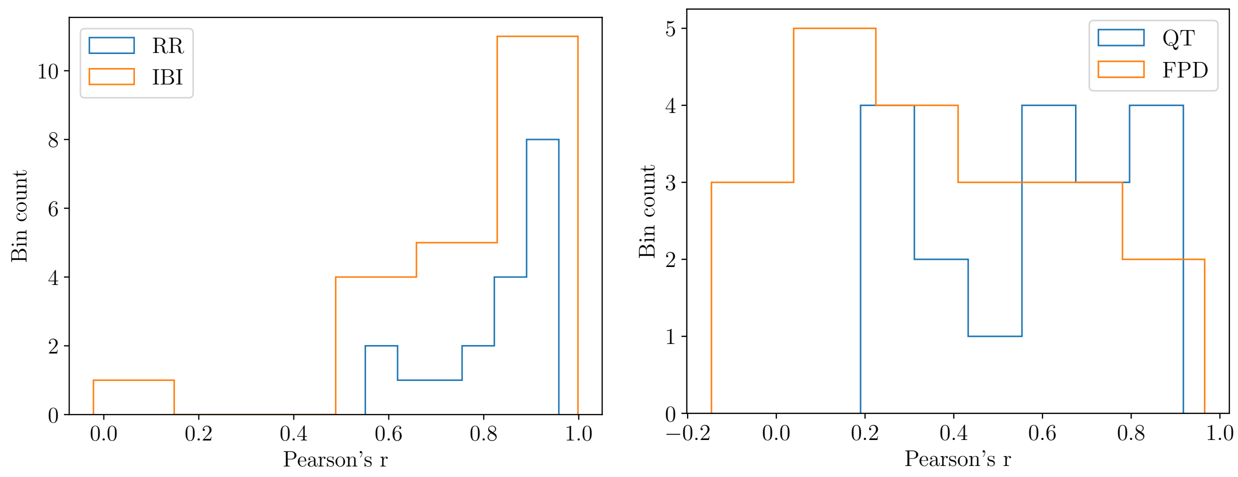

Figure S3: Distribution of the Pearson's correlation coefficient r of RR / IBI (left) and that of QT / FPD (right).

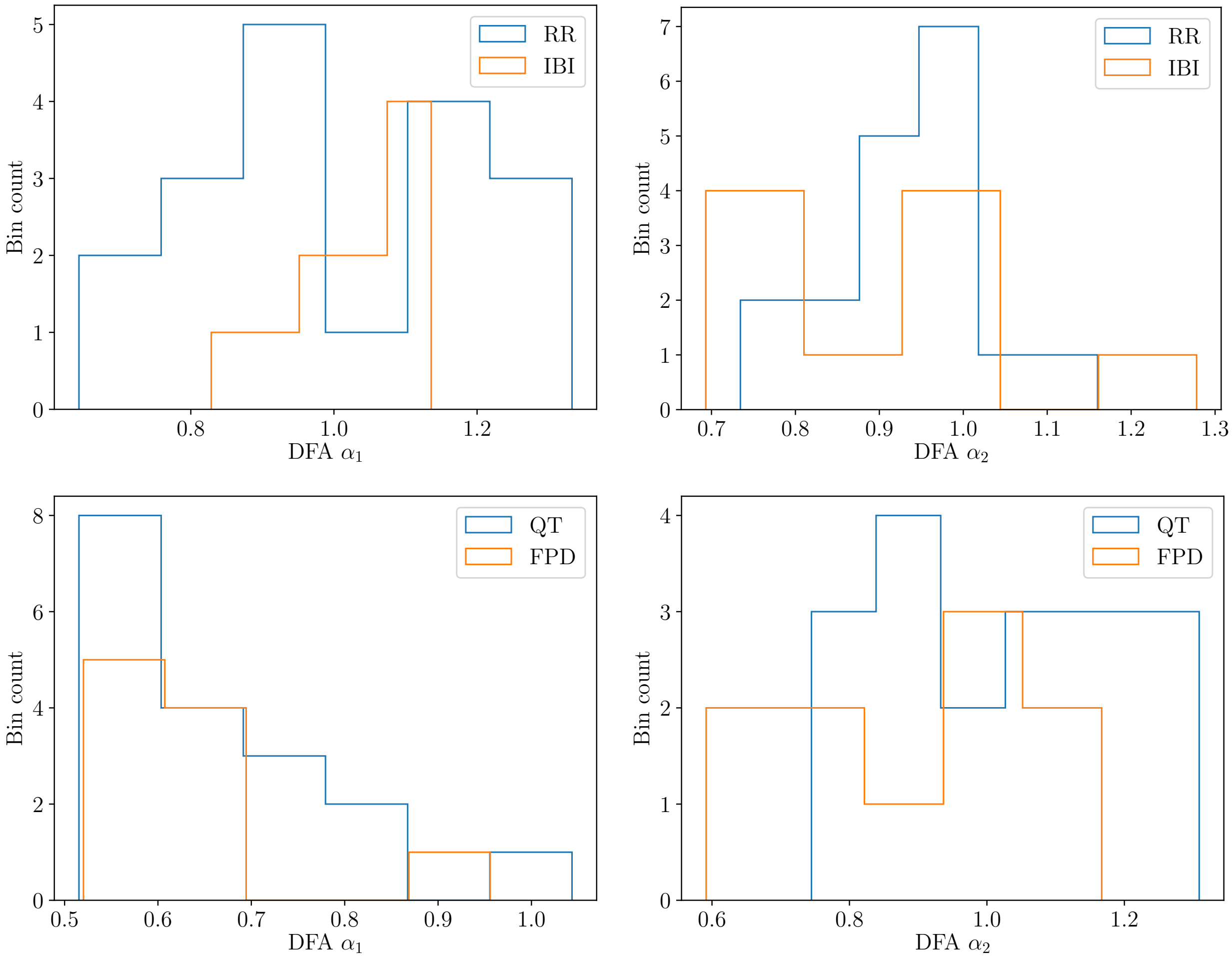

Figure S4: Distribution of DFA scaling exponents α_1 (left) and α_2 (right) for RR and IBI (top) and QT and FPD (bottom).

3 Standard HRV measures of IBI and FPD

Here we report the standard time- and frequency-domain measures that are often used to assess HRV. We chose the measures defined in Table S1.

Table S1: Standard HRV measures and their definitions [1].

AVNN	Average of all NN intervals
SDNN	Standard deviation of all NN intervals
rMSSD	Square root of the mean of the squares of differences between adjacent NN intervals
pNN50	Percentage of differences between adjacent NN intervals that are greater than 50 ms
TOTPWR	Total spectral power of all NN intervals up to 0.04 Hz
ULF	Total spectral power of all NN intervals up to 0.003 Hz
VLF	Total spectral power of all NN intervals between 0.003 and 0.04 Hz
LF	Total spectral power of all NN intervals between 0.04 and 0.15 Hz
HF	Total spectral power of all NN intervals between 0.15 and 0.4 Hz
LF/HF	Ratio of low to high frequency power

The results are summarised with min-max, median, and interquartile range for each measure in Table S2 and S3. The results were obtained with the HRV toolkit software package from PhysioNet [1]. IBI and FPD time series were preprocessed according to the Methods section in the manuscript and used as input to the HRV toolkit program.

Table S2: Standard HRV measures for IBI, the cellular equivalent of RR intervals.

IBI measures	min-max	median	(Q1-Q3)
AVNN (ms)	703 - 2383	**1400**	(1178 - 1745)
SDNN (ms)	4.6 - 241.7	**36.0**	(11.6 - 54.2)
rMSSD (ms)	1.21 - 108.56	**16.25**	(4.45 - 32.36)
pNN50 (%)	0.00 - 58.18	**0.54**	(0.00 - 9.04)
TOTPWR (ms^2)	22.4 - 72827.8	**1392.3**	(137.2 - 3229.2)
ULF (ms^2)	18.7 - 63155.9	**354.9**	(62.0 - 3035.8)
VLF (ms^2)	2.8 - 5086.3	**173.8**	(16.6 - 728.6)
LF (ms^2)	0.5 - 3654.3	**56.7**	(13.3 - 413.3)
HF (ms^2)	0.3 - 2018.7	**51.4**	(6.3 - 219.6)
LF/HF (n.u)	0.26 - 3.32	**1.52**	(0.68 - 2.02)

Table S3: Standard HRV measures for FPD, the cellular equivalent to QT intervals. Note that NN intervals are consecutive FPDs.

FPD measures	min-max	median	(Q1-Q3)
AVNN (ms)	394 - 1529	**902**	(764 - 982)
SDNN (ms)	9.4 - 177.5	**23.9**	(15.6 - 35.7)
rMSSD (ms)	5.40 - 127.70	**23.65**	(17.10 - 41.27)
pNN50 (%)	0.00 - 64.01	**4.79**	(0.44 - 22.97)
TOTPWR (ms^2)	77.1 - 39262.6	**470.0**	(168.2 - 1152.5)
ULF (ms^2)	3.9 - 28497.0	**92.8**	(34.6 - 290.1)
VLF (ms^2)	11.8 - 3812.1	**38.8**	(22.1 - 182.5)
LF (ms^2)	7.5 - 1885.7	**63.6**	(22.4 - 126.7)
HF (ms^2)	8.6 - 5067.8	**143.7**	(43.9 - 358.0)
LF/HF (n.u)	0.19 - 0.88	**0.48**	(0.44 - 0.63)

References

[1] Goldberger, A. L. et al. PhysioBank, PhysioToolkit, and PhysioNet: Components of a new research resource for complex physiologic signals. *Circulation* **101**, e215-e220; 10.1161/01.CIR.101.23.e215 (2000).

[2] Peng, C.-K. et al. Mosaic organization of DNA nucleotides. *Phys. review e* **49**, 1685 (1994).

[3] Kantelhardt, J. W., Koscielny-Bunde, E., Rego, H. H., Havlin, S. & Bunde, A. Detecting long-range correlations with detrended fluctuation analysis. *Phys. A: Stat. Mech. its Appl.* **295**, 441-454 (2001).
